# The Neural Odyssey: Unveiling the Potential of Neuroscience–ICT Convergence

**DOI:** 10.3390/biology12060840

**Published:** 2023-06-09

**Authors:** Daniele Giansanti

**Affiliations:** Centre Tisp, Istituto Superiore di Sanità, 00161 Roma, Italy; daniele.giansanti@iss.it; Tel.: +39-06-49902701

The integration of neuroscience and information and communication technology (ICT) has ignited a profound transformation in the understanding of the complex biology of the brain and neural system. This interconnection highlights a powerful synergy, and calls for the determination of bipolar correspondence between the two scientific fields, from two angles:

- On the one hand, developments in neuroscience are aiding advances in novel ICT tools and vice versa.
- On the other hand, ICT is providing (also by means of informatics imitation of the neural decisional mechanism) new opportunities to explore the intricacies of biological systems (including the brain).

Neuroscience, as it is well known, focuses on knowing the architecture, intricacies, and complexities of the nervous system and brain. This scientific field has made significant progress in investigating the mechanisms directing cognition, perception, and behavior [1,2,3]. Traditional methodologies such as brain imaging, electrophysiology, and molecular biology have contributed to the accumulation of biological and medical knowledge about the neural system’s and brain’s architecture and functionality [4]. However, the integration of neuroscience with information and communication technology (ICT) is sparking a new era of investigation, enabling scientists to delve deeper into the complex biological mechanisms of the brain and central nervous system [5].

The field of neuroimaging, for example, has particularly benefited from this convergence [6]. By combining ICT with advanced imaging techniques, such as functional magnetic resonance imaging (fMRI), researchers can now capture the dynamic activity of the brain with unprecedented precision. fMRI reveals metabolic activity across different brain regions, providing valuable insights into the neural basis of emotions, perceptions, motion, and memorizations [7]. This integration of biology and technology has allowed researchers to visualize the complex work of neural networks in action.

Moreover, the integration of biology and ICT has revolutionized the analysis and interpretation of vast amounts of sophisticated biological data. High-performance computing, artificial intelligence (with different components), and innovative data analytics have equipped researchers with powerful tools to interpret form a microbiological point of view the processes underlying the central nervous system’s and brain’s complexity [8]. This convergence has opened to the explorations in the new emerging fields of proteomics, genomics, and connectomics [9,10].

Additionally, the fusion of biology and ICT has given rise to the field of neuroinformatics [11], which combines ICT tools and techniques/technologies with biological knowledge derived from neuroscience research, establishing a unified framework for data sharing, analysis, and collaboration. Comprehensive databases have been created, representing an important meeting point for research activities of all the world.

This integration of ICT with neuroscience fosters cross-disciplinary investigations, accelerates discoveries, and promotes a deeper understanding of the biological mechanisms of the brain and neural systems.

Today:

- There are countless ICT devices that interface with neuroscience.
- Likewise, there are countless ICT tools that allow biology to be addressed, for example via a neuro-computational approach.

*The application of information and communication technology (ICT) in neuroscience has led to significant advancements, for example, in the design of biomedical interfaces* [12,13,14]. These include the development in wireless EEG devices, allowing researchers to measure brain activity in real time even in uncontrolled environments. ICT has also allowed for the creation of brain stimulation devices. Some important examples are transcranial magnetic stimulation (TMS) and deep brain stimulation (DBS). These are useful in the investigation of brain activity, and in facilitating medical and biological learning to face and treat several neurological disorders. Virtual reality (VR) devices have become useful for the investigation of brain feedback to visual stimulation. Motion sensors, such as electro-goniometers, accelerometers, and gyroscopes, integrated thanks to proper ICT, are used to record body motion, which is particularly useful in the study of the relationships between movement disorders and the brain (e.g., in Parkinson’s disease). Additionally, developments in specific ICT have given rise to brain–computer interfaces (BCIs), which are creating big expectations for direct communication between computers and humans. These technological advancements, in summary, have expanded researchers’ capabilities in studying brain activity, understanding neurological disorders, investigating cognitive processes, improving brain–device interactions, and allowing for the development of new therapies for neurological diseases.

*From another point of view, ICT tools also allow biology to be tackled* via *high-performance informatics tools, such as neuro-computational technologies* [15,16].

Key examples include high-speed DNA sequencing, which has allowed for the decoding of the human genome and the exploration of genetic variants linked to neurological conditions. The integration of computer science and biology using bioinformatics tools has facilitated the analysis of large genomic, proteomic, and transcriptomic datasets, leading, for example, to the detection of the genes involved in brain or nervous system function and neurological pathologies. Enhanced cellular-level imaging tools, such as fluorescence microscopes, have allowed for the investigation of brain cytology, architecture, and functions from a molecular point of view as well. Simulations using ICT (based, for example, on high-performance computers) have helped researchers to understand neural mechanisms and forecast behaviors based on dissimilar settings. Additionally, the design of wide biological data banks, thanks to ICT, has facilitated data sharing, cooperation, and the deep analysis of proteins, genetics, and further biological information. In summary, ICT has revolutionized investigation and analysis in biological data analysis, computational simulation, architecture modeling, and cellular, tissue, and functional imaging.

Some studies have addressed the importance of ICT in neuroscience from a general point of view without going into the ICT tools in detail, but these limit themselves to the two generic components of information and communication. A quick overview on PubMed using the search key, as shown in Box 1, shows 55 papers starting from 2003, whereby 69.1% of which were published in the last ten years, highlighting an acceleration in research interest in this area [17].

Box 1 **The first proposed composite key**

*(neuroscience [Title/Abstract]) AND ((ICT[Title/Abstract]) OR (high technology [Title/Abstract]) OR (digital technology [Title/Abstract]) OR (communication technology[Title/Abstract]) OR (information technology[Title/Abstract]))*

If the development of a specific sector of ICT in the field of neuroscience is then examined in detail, one realizes how, recently, the growth has been terrific, given that studies in this area are now very numerous. Carrying out a search on PubMed related to the studies of AI (ICT tool) in neuroscience, we found 317 articles (see Box 2 for the proposed composite key) [18]. Among these, we also found 116 reviews, a sign of an important gradual stabilization of the topics of interest. The growth in interest in this scientific field is pseudo-exponential. Considering the history, the interest surrounding this subject dates back to 1987. Considering the last ten years, there have been 288 papers, equal to 90.9%. Considering papers from 2020, we found 197, equal to 62.1%. All of this highlights a rapid acceleration in research in this field over the past 10 years, acceleration that has become terrifying in the period associated with the COVID-19 pandemic. Similar examples can be found in other ICT sectors.

Box 2 **The second proposed composite key**

*(neuroscience [Title/Abstract]) AND (artificial intelligence [Title/Abstract])*

This junction between ICT and neuroscience, with the introduction of new technologies such as artificial intelligence, is also generating important questions in the domain of ethics and employment regulation in some ICT sectors, and therefore there has been growth in the attention paid to the so-called *neuro-rights* [19,20].

Everything that has been reported thus far highlights the interest in developing an initiative for discussion and scientific comparison in this area.

This Special Issue, entitled “*The Convergence of Neuroscience and ICT: From Data to Insights*” [21], which acts as a forum of knowledge in this rapidly accelerating field and a link between ICT and neuroscience, addresses both basic and applied research fields, and does not neglect ethical and regulatory issues.

*In conclusion, the convergence of neuroscience and information and communication technology has two main contributions*: On the one hand, it has breathed new life into our exploration of the brain’s biology. By merging biology and technology, researchers can now peer into the brain’s inner workings with unprecedented clarity, revealing the mesmerizing dance of neural circuits and unraveling the mysteries of cognition and consciousness. On the other hand, the application of ICT, based, for example, on a neurocomputational approach, also inspired by neural biological systems, is also allowing us to tackle the immense complexity of biological systems, including neurological ones. We hope that common ground [21] in this area will support the scientific community in the furthering of knowledge.
